# Towards Social Media as DTx in Psychiatry? Current opportunities and pitfalls

**DOI:** 10.1192/j.eurpsy.2023.192

**Published:** 2023-07-19

**Authors:** L. Orsolini

**Affiliations:** Department of Neurosciences/DIMSC, Polytechnic University of Marche, Ancona, Italy

## Abstract

**Abstract:**

Software-driven therapeutic interventions aiming at preventing, managing or treating medical or chronic diseases are referred to as Digital Therapeutics (DTx). DTx are developed to target a specific disorder or disease, including mental disorders. DTx are regarded as an emerging class of medicines and, hence, have obtained the approval of the relevant regulatory authorities, based on clinical evidence for the effectiveness similar to conventional medicine supplies and medicines. Social media-based DTx can represent both a means to quantify mental health as well as a source of both positive and negative interactions, including a source of social support for many who have been socially isolated and lonely. Recent researches using monitored social networks as interventions has shown promise in youth with several mental health issues, despite it is noteworthy that social media are not without risks.

**Disclosure of Interest:**

None Declared

